# Maternal Immunization with Pneumococcal Surface Protein A Protects against Pneumococcal Infections among Derived Offspring

**DOI:** 10.1371/journal.pone.0027102

**Published:** 2011-10-31

**Authors:** Masamitsu Kono, Muneki Hotomi, Susan K. Hollingshead, David E. Briles, Noboru Yamanaka

**Affiliations:** 1 Department of Otolaryngology-Head and Neck Surgery, Wakayama Medical University, Wakayama-city, Wakayama, Japan; 2 Department of Microbiology, University of Alabama at Birmingham, Birmingham, Alabama, United States of America; Monash University, Australia

## Abstract

Pathogen-specific antibody plays an important role in protection against pneumococcal carriage and infections. However, neonates and infants exhibit impaired innate and adaptive immune responses, which result in their high susceptibility to pneumococci. To protect neonates and infants against pneumococcal infection it is important to elicit specific protective immune responses at very young ages. In this study, we investigated the protective immunity against pneumococcal carriage, pneumonia, and sepsis induced by maternal immunization with pneumococcal surface protein A (PspA). Mother mice were intranasally immunized with recombinant PspA (rPspA) and cholera toxin B subunit (CTB) prior to being mated. Anti-PspA specific IgG, predominantly IgG1, was present at a high level in the serum and milk of immunized mothers and in the sera of their pups. The pneumococcal densities in washed nasal tissues and in lung homogenate were significantly reduced in pups delivered from and/or breast-fed by PspA-immunized mothers. Survival after fatal systemic infections with various types of pneumococci was significantly extended in the pups, which had received anti-PspA antibody via the placenta or through their milk. The current findings strongly suggest that maternal immunization with PspA is an attractive strategy against pneumococcal infections during early childhood. (191 words)

## Introduction

*Streptococcus pneumoniae* frequently colonize the nasopharynx asymptomatically. Especially following viral infections, *S. pneumoniae* are responsible for a significant proportion of bacterial infectious diseases such as meningitis, otitis media, bacteremia, and pneumonia [1]. The high incidence of pneumococcal disease starts in the neonatal period and peaks around the first birthday. Efforts have focused on the protection of children against pneumococcal infections by immunization with vaccines.

The current 23-valent pneumococcal polysaccharide vaccine (PPV) is efficacious in adults [2]. However, this polysaccharide-based vaccines evoke little or no immune response in infants younger than 2 years of age because of the weak immunogenicity of its T cell independent polysaccharides [3], [4]. Protein-conjugated polysaccharide vaccines have been considered as an alternative means to induce protective immunity in infants and children [5], [6]. Human trials of a 7-valent polysaccharide conjugate vaccine (PCV7) showed the capability to elicit solid protection against invasive pneumococcal infection in children [7]–[11]. However, PCV7 is not protective against strains with capsular types/groups not present in the vaccine [12], [13]. Shortly after the vaccine was licensed, reports of serotype replacement began to appear [14]–[16]. Efforts to circumvent the problem of serotype replacement have included expanding the number of polysaccharides in the vaccine but this will not necessarily avoid the problem of subsequent serotype replacement [6], [17], [18].

Furthermore, children younger than 2-years old usually have low levels of IgG serum antibody to pathogen-specific antigens; a results of age-related immaturity of immune responses [19], [20]. The recurrent bacterial infections are thought to be in part due to the subnormal levels of serum IgG antibody against causative pathogens due to age-related immaturity [20]–[23]. Virolainen et al showed that children who were infected most frequently with pneumococci had the lowest titer of antibody to PspA among children with invasive pneumococcal infections [24]. Simell et al have made a similar observation showing that higher salivary antibody levels to PspA are associated with a lower rate of pneumococcal otitis media [25]. The need for protein-based pneumococcal vaccines and their ability to protect against pneumococcal infections during infant period has been further emphasized by studies demonstrating a recent rapid increase in both the prevalence and levels of resistance of multiple antimicrobial resistant pneumococci [16]. Maternal immunization with PPV is reported to reduce acute lower respiratory infections in infants [26], [27].

Pneumococcal surface protein A (PspA) is a promising candidate for inclusion in a cost-effective protein-based pneumococcal vaccine. PspA is an exposed virulence factor present in virtually all pneumococcal strains. It is a highly immunogenic antigen and affects host-pathogen interactions by inhibiting complement activation by the classical and alternative pathways [28]–[30]. PspA can elicit an antibody response that enhances complement deposition and protects against nasal carriage, pneumonia, and bacteremia in animal models [31]–[33]. Moreover, a human trial showed an increase in specific anti-PspA immunoglobulin G (IgG) levels after immunization with rPspA. Sera from the humans immunized with rPspA were able to passively protect mice against otherwise fatal challenge with various pneumococcal strains [34], [35].

Our preliminary study evaluated the efficacy of maternal immunization with rPspA for protecting against lethal systemic pneumococcal infections [36]. In the current study, we further evaluated the relative roles of placental and milk/colostrum derived antibody in the protection against pneumococcal invasive disease and carriage in mouse pups following maternal immunization with rPspA. Prospective mother mice were intranasally immunized with rPspA. Antibody levels in the breast milk and serum of the mothers were measured and the transmission of antibody and protection to the pups was evaluated by challenging the offspring of mothers and the offspring of mice that were fostered on immunized and non-immunized mothers.

## Results

### Anti-PspA specific antibodies in sera and milk of mother mice

**Figure 1:**
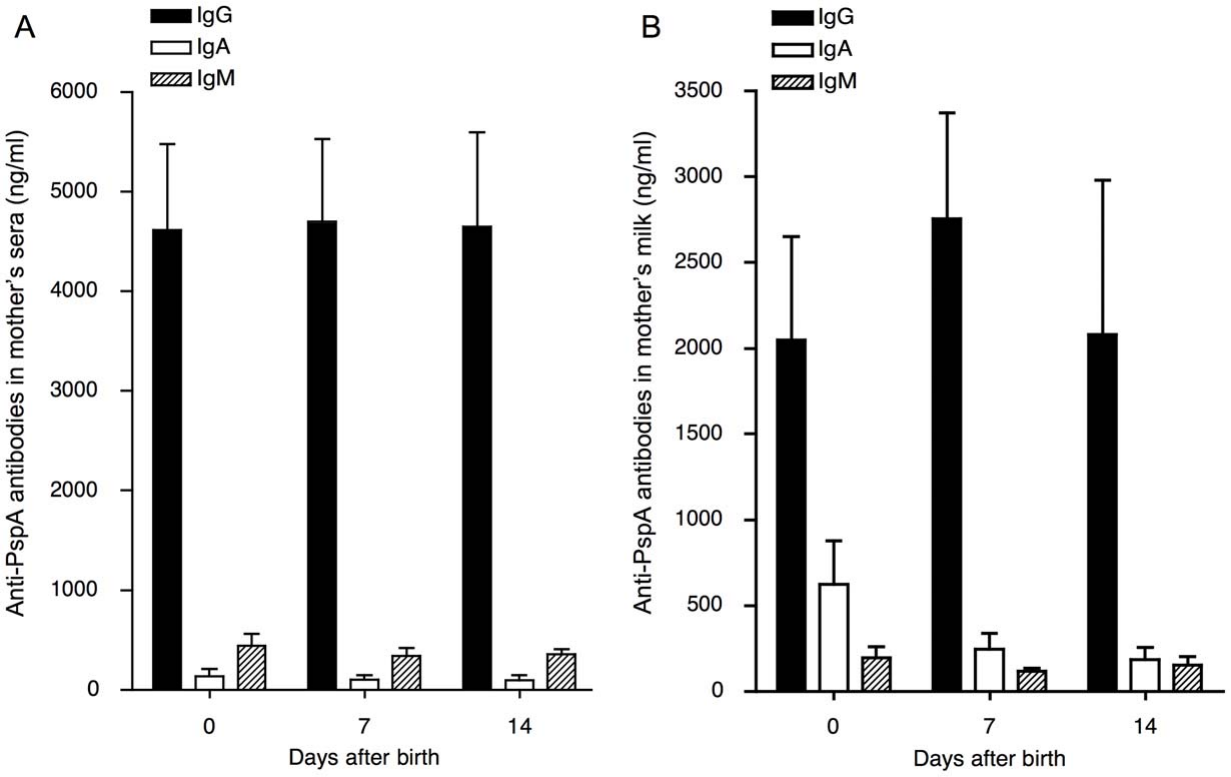
Anti-PspA specific antibodies in sera and milk of mother mice. Female mice were intranasally immunized twice each week with 1 µg of rPspA and 4 µg CTB for first 2 weeks and with 1 µg rPspA alone for the last week. The levels of anti-PspA specific IgG, IgA and IgM antibodies in sera (A) and breast milk (B) were determined by PspA-specific ELISA on day 0, 7 and 14 after the birth. The values shown are the mean ± S.E. concentrations (ng/ml) taken from PspA-immunized mother (n = 16) and sham-immunized mother (n = 14). The levels of anti-PspA specific antibodies in sera and breast milk from sham-immunized mice were below the limit of detection.

The levels of anti-PspA specific antibodies in sera of mother BALB/cByJ mice were evaluated on day 0, 7, and 14 after they gave birth (Fig. 1A). Anti-PspA specific IgG in sera of pups was present at the birth and maintained during nursing periods among PspA-immunized mother mice. Anti-PspA specific IgA and IgM were also identified among PspA-immunized mother mice although the levels of anti-PspA specific IgA and IgM in sera were relatively low rather than anti-PspA specific IgG. The levels of anti-PspA specific antibodies in sera among pre-immunized mother mice and sham-immunized mother mice were below the detections limit (data not shown).

The levels of anti-PspA specific antibodies in breast milk from mother mice were also evaluated on day 0, 7, and 14 after the birth (Fig. 1B). In breast milk higher levels of IgG PspA-specific antibody were detected relative to IgA or IgM specific antibody. The levels of anti-PspA specific antibodies in breast milk among pre-immunized mother mice and sham-immunized mother mice were below the detections limit (data not shown).

### IgG subclasses of antibody to PspA in sera and milk of mother mice

**Figure 2:**
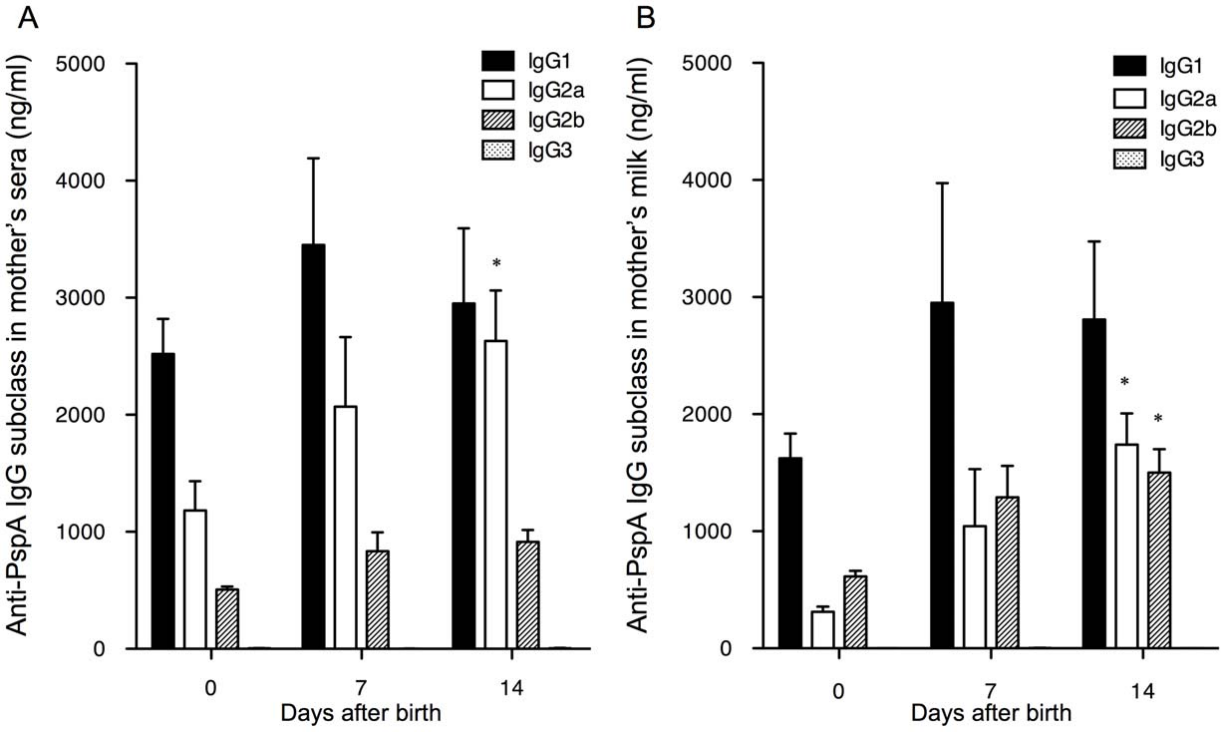
Anti-PspA specific IgG subclasses in sera and milk of mother mice. Female mice were intranasally immunized twice each week with 1 µg of rPspA and 4 µg CTB for first 2 weeks and with 1 µg rPspA alone for the last week. The levels of anti-PspA specific IgG1, IgG2a, IgG2b and IgG3 antibodies in sera (A) and breast milk (B) were determined by PspA-specific ELISA on day 0, 7 and 14 after the birth. The values shown are the mean ± S.E. concentrations (ng/ml) taken from PspA-immunized mother (n = 16) and sham-immunized mother (n = 14). The mean values of IgG1/IgG2a antibody to PspA in the sera of the individual mother's sera were 3.1, 2.1 and 1.1 for day 0, 7, and 14, respectively. The mean IgG1/IgG2a anti-PspA values for the individual mother's milk samples were 5.7, 6.5, and 1.7 for day 0, 7, and 14, respectively. The levels of anti-PspA specific IgG subclasses in sera and breast milk from sham-immunized mice were below the detections limit. * *p*<0.05 when compared with mice at day 0 by ANOVA test or Kruskal-Wallis test with Dunn's multiple comparison test. n.d. not determined.

On the day of birth (day 0) the predominant IgG subclass of IgG antibody to PspA in sera from PspA-immunized mother mice was IgG1, followed by IgG2a and IgG2b (Fig. 2A). The levels of anti-PspA specific IgG2a gradually increased from day 0 to day 14 (*p*<0.05), while the levels of anti-PspA specific IgG1 and IgG2b did not change significantly. The mean IgG1/IgG2a ratio in the sera of individual mice gradually decreased from 3.1 on day 0 to 1.1 on day 14 (*p*<0.05). The levels of IgG3 were below the detection limit.

In the breast milk, IgG1 was also the predominant anti-PspA specific IgG subclass followed by IgG2a and IgG2b from PspA-immunized mother mice (Fig. 2B). The levels of anti-PspA specific IgG2a and IgG2b increased from day 0 to day 14 (*p*<0.05), while IgG1 did not change. The mean IgG1/IgG2a ratio in breast milk from individual mice gradually decreased from 5.7 on day 0 to 1.7 on day 14 (*p*<0.05). The levels of IgG3 were below the detection limit.

### Anti-PspA specific antibodies in sera of offspring

**Figure 3:**
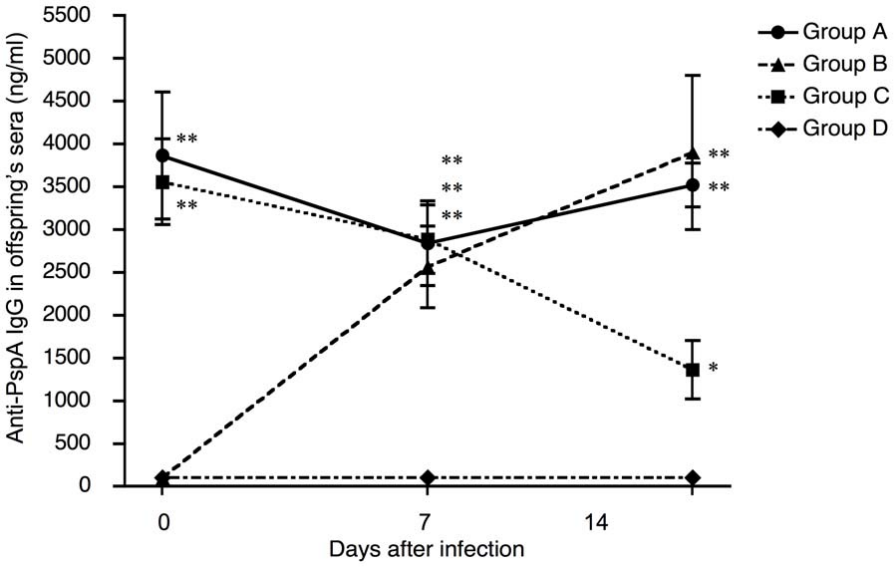
Anti-PspA specific antibodies in sera of offspring. The levels of anti-PspA specific IgG in sera of offspring were determined by PspA-specific ELISA at days 0, 7, and 14 after the birth. Group A mice were the offspring delivered from PspA-immunized mothers and breast-fed by the same mothers (n = 26). Group B mice were offspring from sham-immunized mothers and breast-fed by PspA-immunized mothers (n = 22). Group C mice were offspring from PspA-immunized mothers and breast-fed by sham-immunized mothers (n = 27). Group D mice were offspring from sham-immunized mother and breast-fed by the same mother (n = 18). The values shown are the mean ± S.E. concentrations (ng/ml). * *p*<0.05 and ** *p*<0.01 when compared with offspring in Group D by ANOVA test with Dunn's multiple comparison test.

The changes of anti-PspA specific IgG in the sera of offspring were evaluated on day 0, 7, and 14 after the birth (Fig. 3). Offspring delivered from PspA-immunized mothers (Group A and Group C) had high levels of anti-PspA specific IgG in sera at birth. The levels of anti-PspA specific IgG in sera from offspring of immune mothers that were breast-fed by PspA-immunized mother mice (Group A) were maintained at the high levels on day 7 and day 14. In contrast the levels of anti-PspA specific IgG in sera from offspring of immune mothers who were breast-fed by sham-immunized mother mice (Group C) rapidly declined after the birth. On the other hand, offspring delivered from sham-immunized mother mice (Group B and Group D) did not have anti-PspA specific IgG in sera at the birth. The PspA-specific IgG in sera from offspring of sham-immune mothers that were breast-fed by PspA-immunized mothers (Group B) gradually increased and reached levels similar to those of Group A on day 7 to day 14. The control offspring (Group D) from sham-immunized mothers who were nursed on sham-immune mothers did not have anti-PspA specific IgG in their sera. Anti-PspA specific IgA and IgM were not detected in sera of all offspring.

### Anti-PspA specific IgG subclasses in sera of offspring

**Figure 4:**
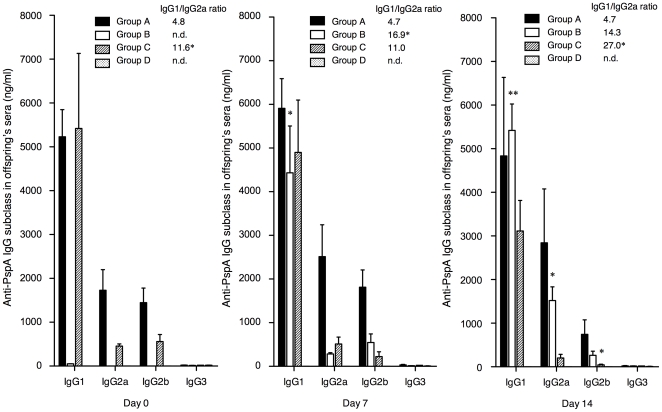
Anti-PspA specific IgG subclasses in sera of offspring. The levels of anti-PspA specific IgG subclasses in offspring's sera were determined by PspA-specific ELISA on day 0, 7, and 14 after birth. Group A (n = 26), B (n = 22), C (n = 27), and D (n = 18) mice were the same mice as described in figure 3. The values shown are the mean ± S.E. concentrations (ng/ml). The mean values of IgG1/IgG2a ratio were also shown. * *p*<0.05 and **p<0.01 are for comparisons with offspring in Group D for PspA-specific IgG subclasses or with offspring on day 0 for the IgG1/IgG2a ratio by ANOVA test with Dunn's multiple comparison test.

The levels of anti-PspA specific IgG subclass in sera of offspring were also evaluated on day 0, 7, and 14 after the birth (Fig. 4). The predominant IgG subclass in sera of offspring in all groups was IgG1 followed by IgG2a and IgG2b at the birth. In all offspring, the levels of IgG3 were below the detection limit.

In Group A, the levels of IgG1 and IgG2a were not changed during the period from day 0 to day 14. In contrast to the results of group A mothers' sera, the mean IgG1/IgG2a ratio calculated from the individual pup sera also did not change during the period from day 0 to day 14. This indicates that IgG2 antibody was less efficiently transported to the progeny by nursing than the IgG1 antibody. In Group B, offspring did not have anti-PspA specific IgG in sera at the birth. The levels of anti-PspA specific IgG1 in sera gradually increased and reached the similar levels to those of the offspring in Group A on day 7 to day 14 (*p*<0.05 and *p*<0.01, respectively). The mean IgG1/IgG2a ratio for group B was higher on day 7 than that of Group A (*p*<0.05). On day 14 the mean IgG1/IgG2a ratio for Group B was higher than that of Group A, but the difference was not statistically significant. In Group C, anti-PspA specific serum IgG1 was predominant on day 0 and the levels did not change during the period from day 0 to day 14. The IgG2b levels in the Group C mice gradually decreased from day 0 to day 14. The mean IgG1/IgG2a ratios for Group C mice were higher than those of Group A mice on day 0 and increased from day 7 to day 14 (*p*<0.01).

### Protection against nasal carriage of pneumococci by maternal immunization with PspA among offspring

**Figure 5:**
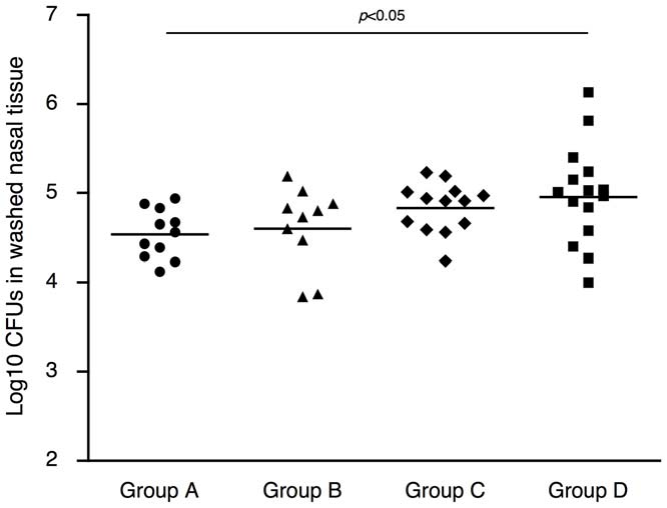
Protection against nasal carriage of pneumococci by maternal immunization with PspA among offspring. Offspring at 7-day-old were intranasally challenged with 1×10^5^ CFU TIGR4 strain (5 µl/mouse) without anesthesia. Two days after challenge, nasal washes and homogenized washed nasal tissues were collected and the numbers of pneumococci colonies were determined. No evidence of protection was observed in CFUs in nasal washes (not shown). Results are shown for CFU in homogenized washed nasal tissue. Each dot represents the Log_10_ CFU/mouse. Each horizontal line depicts the median Log_10_ CFU/mouse. Group A (n = 11), B (n = 10), C (n = 13), and D (n = 15) mice were produced in the same manner as the corresponding groups in figure 3. Group A differed from Group D at *p*<0.05 by Kruskal-Wallis test with Dunn's multiple comparison test.

The carriage density of pneumococci in nasal washes and homogenized washed nasal tissue were evaluated at day 2 after intranasal challenge with 5×10^5^ CFUs of TIGR4 pneumococci. The numbers of CFUs in nasal washes were not different among groups. The median Log_10_ CFUs of pneumococci in nasal washes of Group A, B, C, and D was 4.55, 4.45, 4.24, and 4.49, respectively. On the other hand, the carriage density of the homogenized washed nasal tissue was significantly different among groups (Fig. 5). The median Log_10_ CFUs of pneumococci in nasal tissue of Group A, B, C, and D was 4.56, 4.77, 4.91, and 5.01, respectively. The carriage density of washed nasal tissues of Group A was statistically lower than those of Group D (control) (*p*<0.05). The Log_10_ CFUs of washed nasal tissue of Group B tended to be lower than that of Group D (*p*<0.1). There was no difference in CFUs in nasal tissue of Groups C and D. Thus, maternal immunization with PspA appeared to result in only a modest reduction of nasal colonization of nasal tissue among offspring nursed on immunized dams.

### Protection against lung infection by maternal immunization with PspA among offspring

**Figure 6:**
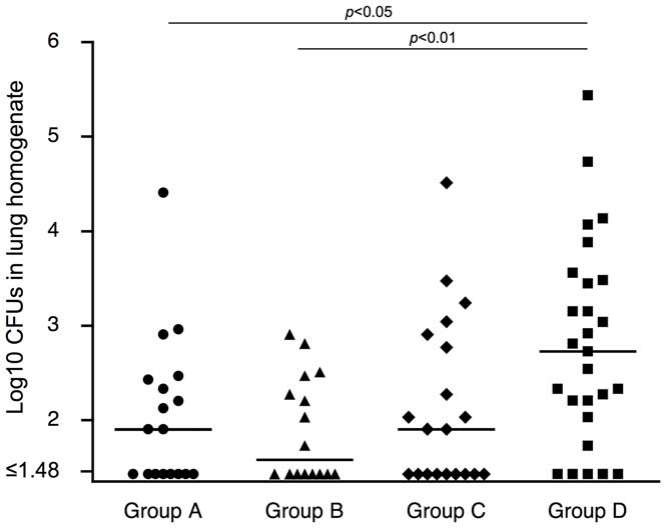
Protection against lung infection by maternal immunization with PspA among offspring. Seven-day-old mice were intranasally challenged with 5×10^5^ CFU TIGR4 strain (10 µl/mouse) with anesthesia. Three days after challenge, lungs were collected and the numbers of pneumococci colonies in the lung homogenate were determined. Each dot shows the Log_10_ CFU/mouse. Each horizontal line shows the median Log_10_ CFU/mouse. Group A (n = 18), B (n = 16), C (n = 20), and D (n = 27) mice were produced in the same manner as the corresponding groups in figure 3. *p*<0.05 and *p*<0.01 are *p*-values for differences between the indicated group and the non-immune mice in Group D by Kruskal-Wallis test with Dunn's multiple comparison test.

Inoculation of 7-day old offspring with a relatively large volume of inoculum intranasally under anesthesia caused enough pneumococci to be aspirated to cause infection of the lung. The 5×10^5^ CFUs of TIGR4 pneumococci in 10 µl sterile Ringer's solution were inoculated intranasally into anesthetized offspring. At 3 days after inoculation, all mice were alive and were euthanized so that the numbers of CFU in their lungs and blood could be determined (Fig. 6). The median Log_10_ CFUs of pneumococci in Group A, B, C, and D was 1.91, 1.58, 1.90, and 2.73, respectively. The mean number of Log_10_ CFUs in lung homogenate in Group A and Group B were each significantly reduced in comparison to Group D (*p*<0.05 and *p*<0.01, respectively). Mice from immunized mothers who were not nursed by immune mothers (Group C) had fewer median CFU than the non-immune Group D mice, this difference was not statistically significant. In all cases there were no CFU or only a few in the blood. As a result, we can be confident that the protection seen was the result of events in the lung and not protection against sepsis. Based on these results immunity achieved through nursing appeared to be especially important for protection in this model.

### Protection against fatal systemic pneumococcal infections among offspring through maternal immunization with rPspA

**Figure 7:**
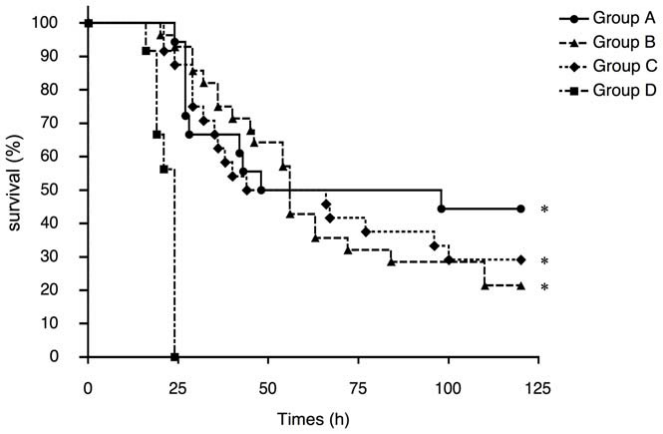
Protection against fatal systemic pneumococcal infections by maternal immunization with PspA among offspring. Offspring at 10-days of age were intraperitonally challenged with 1×10^4^ CFU TIGR4 strain (100 µl/mouse) with anesthesia. After challenge, offspring were monitored for 5 days to determine survival. Group A (n = 24), B (n = 28), C (n = 24), and D (n = 38) mice were produced in the same manner as the corresponding groups in figure 3. Group A mice are offspring delivered from PspA-immunized mothers and breast-fed by the same mothers (n = 24). Group B mice are offspring from sham-immunized mothers and breast-fed by PspA-immunized mothers (n = 28). Group C mice are offspring from PspA-immunized mothers and breast-fed by sham-immunized mothers (n = 24). Group D mice are offspring from sham-immunized mothers and breast-fed by the same mothers (n = 38). * *p*<0.01 when compared with control offspring in Group D by Kaplan-Meier test with Log rank test.

Survival of offspring after intraperitoneal infection with 1×10^4^ CFUs of TIGR4 strain was evaluated. The survival after the otherwise fatal systemic pneumococcal infection was significantly extended in Groups A, B, and C as compared to the sham-immunized Group D controls (*p*<0.01 for each group) (Fig. 7). With this model, transfers of antibody by the placenta and/or nursing were all exhibited significant protection.

In all of the above studies we immunized with TIGR4 derived PspA and challenged with TIGR4 capsular type 4 strain. However, there is some variability in PspA and it is found in two broad serologically cross-reactive families; PspA serologic/sequence family 1 (PspA1) and PspA serologic/sequence family 2 (PspA2) [37]. It is generally recommended that in the development of a human vaccine that one PspA1 protein and one to two PspA2 proteins be used [38]. However, there have also been findings that in some studies that strong cross-protection could be observed between PspA1 and PspA2 families [39]–[43].

**Figure 8:**
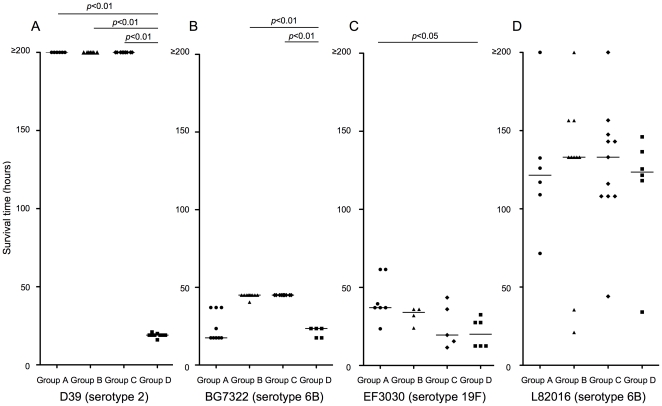
Cross-protection against fatal infections with pneumococcal strains expressing family 1 PspA. As in the prior studies the mother mice were immunized with a rPspA2 of strain TIGR4. Offspring at 10-days of age were intraperitoneally challenged with D39 (PspA1, serotype 2; 50 CFUs/mouse), BG7322 (PspA1, serotype 6B; 20 CFUs/mouse), EF3030 (PspA1, serotype 19F; 5×10^6^ CFUs/mouse), and L82016 (PspA1 serotype 6B, 5×10^6^ CFUs/mouse) in 100 µl with anesthesia. After challenge, the mice were monitored for 10 days to determine the day of death. Group A mice were offspring delivered from PspA-immunized mothers and breast-fed by the same mothers (n = 6 for D39, n = 10 for BG7322, n = 7 for EF3030, n = 6 for L82016). Group B mice were offspring from sham-immunized mothers and breast-fed by a PspA-immunized mothers (n = 7 for D39, n = 11 for BG7322, n = 4 for EF3030, n = 10 for L82016). Group C were offspring from PspA-immunized mothers and breast-fed by sham-immunized mothers (n = 9 for D39, n = 11 for BG7322, n = 5 for EF3030, n = 11 for L82016). Group D mice were offspring from sham-immunized mothers and breast-fed by the same mothers (n = 9 for D39, n = 5 for BG7322, n = 6 for EF3030, n = 6 for L82016). *p*<0.05 and *p*<0.01 for the indicated comparisons with offspring in Group D by Kruskal-Wallis test with Dunn's multiple comparison test are shown.

TIGR4 strain is PspA2 and we evaluated its ability to elicit immunity to four different PspA1 strains. In each case the highest challenge dose was used to reproducibly kill 100% of non-immune control mice. Survival of offspring infected intraperitoneally with PspA1 strain D39 (capsular serotype 2, 50 CFUs/mouse) was significantly extended (*p*<0.01) compare to that of controls. Survival of offspring infected with two other PspA1 strains of EF3030 (capsular serotype 19F, 5×10^6^ CFUs/mouse) and BG7322 (capsular serotype 6B, 20 CFUs/mouse) were weakly extended compared to that of controls (Fig. 8). However, there were no significant differences in survival times among offspring infected the PspA1 strain with L82016 (capsular serotype 6B, 5×10^6^ CFUs/mouse). These finding makes it clear that even when immunization is not with the homologous PspA family it is still sometimes possible to see a protective response in the pups. However, these data are consistent with the expectation that a PspA vaccine should include PspAs of both major PspA families.

## Discussion

During the first few months of life, when the human immune system is still immature, infants depend largely on passively acquired maternal IgG antibodies to protect themselves against invasive pathogens [20]. As in young humans, neonatal and infant mice are more susceptible to pneumococcal colonization and subsequent infection than are adults. Neonatal mouse macrophages show impaired innate and adaptive immune responses to pneumococci, which might explain the increased susceptibility to pneumococcal colonization in vivo [44]. The vaccines used to induce protective antibody early in childhood serve to minimize this window of natural susceptibility, but all such vaccines leave a window of susceptibility during the time that the initial immune response is induced. Immunization of women before pregnancy is a strategy that has been proven to reduce infection risks in mothers and infants for more than one pathogen [45]–[50].

Many recent pre-clinical studies in animals have focused on developing effective mucosal vaccines to combat the susceptibility of children to respiratory bacteria [51]–[53]. Our previous immunization studies using the outer membrane protein P6 of *Haemophilus influenzae* showed that maternal intranasal immunization could induce anti-P6 specific IgG antibody responses in mother's sera and breast milk at birth and that the immune response was maintained for 14 days during the nursing period [54]. Similar to those previous results, our present studies showed that anti-PspA specific antibody predominant in IgG was observed in PspA-immunized mother mice and was transported to their offspring via placenta and breast milk.

Mouse colostrums or breast milk have been reported to contain higher amounts of IgG antibody compared to IgA and IgM antibodies [55]. In mice, IgG antibody in mother's sera is transferred from mother to fetus through placenta by neonatal Fc receptor, FcRn. This antibody is initially collected in the yolk sacs of prenatal mice and rats [56], [57]. Moreover, IgG antibody in breast milk is also transferred from intestine lumen to systemic circulation in neonate mice [56], [57]. This transport of IgG antibody is mediated by FcRn expressed in the intestine of mice and rats [58]–[61]. The idea that IgG may cross epithelial barriers by receptor-mediated transcytosis in humans and other animals represents a novel concept in mucosal immunology. Recent studies have also demonstrated receptor-mediated IgG transport demonstrated across the lung of mice [62]. The FcRn expressing bronchia epithelial cells transports IgG across the mucosal surface of lung from lumen to serosa. The neonatal FcRn mediates the transport of IgG across polarized epithelial cells lining mucosal surface [63], [64]. Furthermore, not only is IgG transmitted to progeny, but functional maternal immunoglobulin secreting cell or B cells can also be transferred to the neonate in both mouse and human [65], [66].

In the present study of antibody to PspA, we further evaluated the protection of offspring against nasal carriage, lung infection, and fatal sepsis caused by pneumococci. Nasopharyngeal colonization is the initial step in the pathogenesis of infection caused by *S. pneumoniae*. Since carriage is considered to precede the development of subsequent fatal pneumococcal invasive diseases, the protection against carriage can also protect against subsequent disease [67].

The numbers of pneumococci colonizing closely associated with the nasal tissue were reduced by maternal immunization with PspA. On the other hand, the numbers of pneumococci washed from nasal surfaces were not different among groups (data not shown). Our earlier study demonstrated that during nasal colonization of mice with pneumococci, the majority of the colonizing pneumococci are tissue-associated and of the opaque phenotype [68]. This observation suggests that the opaque pneumococci may have either invaded the nasal tissue or may be sequestered in deep crypts and are not removed by the nasal wash. While secretory-IgA (SIgA) antibodies play an important role in the protection against nasopharyngeal colonization of pneumococci [69], SIgA make little sense for protecting against pneumococci once they have invaded the nasal tissues [69].

Ferreira et al reported that the reduction of nasal colonization was strongly associated with increased levels of IgG2a complement fixing antibody and lower levels of IgG1 antibody which had less complement fixing activity [70], [71]. As a consequence, lower IgG1/IgG2a ratios were also correlated with lower levels of colonization [70], [71]. In this study, IgG1/IgG2a ratio was decreased on day 0 to 14 in both mother's sera and breast milk. As for the offspring's sera in our study, the anti-PspA specific IgG2a antibodies were increased in sera from offspring in Group A where immunized mothers nursed their own pups. A balanced IgG1/IgG2a antibody response was maintained in the Group A offspring over time. The fact that the only group that showed even a hint of protection against colonization was group A, which had the highest relative concentrations of IgG2a as compared to IgG1, is consistent with the earlier published observations from experiments with adult mice [71]. Our failure to see stronger protection against carriage, may in retrospect be due the fact that our mice were sacrificed only 2 days post challenge. In most adult mouse studies sacrifice at 5-day, 7-day, or later time points, which may have permit the cumulative actions of immunity over time may have had larger effects on colonization.

As compared to colonization, where the protective effects were quite modest if at all, maternal immunization was clearly protectected the offspring against pneumococcal lung infections and fatal sepsis. The absence of bacteremia and sepsis in the aspiration-pneumonia infection model might suggest that the observed protection against lung infection results from direct protection in the lung, rather than just being realized through a protection against septicemia. Offspring delivered from mother mice immunized intranasally with PspA were protected from systemic pneumococcal infections. Intranasal immunization with PspA has been shown to protect adult mice against pneumonia and fatal sepsis and occasionally against nasal carriage [72], [73]. The present studies have shown that similar immunizations of mother mice can protect progeny in these models and that the protection is to some extent independent of the PspA family of the challenge strain.

While significant protection was seen against infection of TIGR4 PspA2 strain by maternal immunization with homologous PspA2, we observed strong portection against invasive infection with only one of four PspA1 challenge strains. The current findings caution that for protein vaccines one must take care to include PspAs representative of both major PspA families: PspA1 and PspA2. Darrieux M. et al. demonstrated that a chimeric fusion protein of PspA1 and PspA2 fragment extend protection against pneumococcal infection with strains bearing diverse PspA fragments [74].

Although the immune systems are different between mice and human being, maternal intranasal immunization can induce specific immune responses in mothers and that this immunity is effectively transferred to their infants. The findings strongly suggest that maternal mucosal (intranasal) immunization would be an attractive procedure to elicit early immunity against *S. pneumoniae* infections among young children. It is anticipated that this immunity is mediated by transplacental immunoglobulin (Ig) transferred during pregnancy and via breast milk. Naturally acquired IgA and IgG antibodies to PspA have been shown to be transferred from mother to child and to protect against early pneumococcal infections. Baril et al reported that transplacental transfer of IgG antibody to PspA was more efficient with IgG1 than IgG2 [75]. This finding is not inconsistent with a potential protective role of transplacental IgG in humans since human IgG1 and IgG2 are both highly complement fixing like IgG2 in the mouse [76]–[78]. Mouse IgG1 is poorly complement fixing and this observation may explain its weaker association with protection in this paper and in earlier studies [70].

Thus, infants may be able to be protected by prior immunization of their mothers. If pneumococcal protein-based vaccines are found to be efficacious, the direct correlation of the magnitude of the mother's IgG to PspA and that of the infant could be an indicator that the immunization of women of childbearing age with protein pneumococcal vaccine could protect their infants from pneumococcal disease. A clinical trial could be designed to test the potential of immunization of young adult women or infants with a combination of pneumococcal proteins as an experimental vaccination approach to prevent against invasive pneumococcal disease.

In conclusion, the current findings suggest that maternal intranasal immunization would be an attractive procedure against pneumococcal infections among during childhood because the transport of the specific antibody to the neonate can be expected to occur through both the placenta and mother's milk.

## Materials and Methods

### Bacterial strains

*S. pneumoniae* strains TIGR4 (serotype 4, PspA2), D39 (serotype 2, PspA1), EF3030 (serotype 19F, PspA1), L82016 (serotype 6B, PspA1), and BG7322 (serotype 6B, PspA1) used in this study. All of *S. pneumoniae* strains were grown in Todd-Hewitt broth with 0.5% yeast extract (THY) at 37°C until mild log-phase and stocked in aliquots at known CFU concentrations in THY broth containing 10% glycerol at −80°C until use for infections.

### Recombinant PspA

rPspA for immunization was PspA2/TIGR4 including α-helical region [37]. Briefly, an internal gene fragment of *pspA* was amplified by polymerase chain reaction from *S. pneumoniae* strain TIGR4. The amplified gene fragment of the expected sizes were sub-cloned by TOPO TA Cloning Kit (Invitrogen Inc., Carlsbad, CA, USA) and then cloned to the pET20b vector (Novagen Inc., Madison, WI, USA) incorporating between *Nco*I and *Xho*I sites. The pET20b vector containing *pspA* fragment was transformed into the *E. coli* strain BL21 (DE3) for protein production. Expression of rPspA was induced with 1 mM isopropylthio-ß-D-galactoside (IPTG) for 2 h. The six-histidine-tagged rPspA was purified by nickel affinity chromatography.

### Immunization

Four-week-old BALB/cByJ female mice were maintained under specific pathogen-free condition. They were immunized twice each week with 1 µg of rPspA mixed with 4 µg cholera toxin B subunit (CTB) (List Biological Labs, Campbell, CA, USA) on the Mondays and Fridays of 3 consecutive weeks [32]. During the first two weeks the immunization included CTB. During the last week, the two immunizations contained antigen alone. Control mice received only CTB for the first 2 weeks and only saline for the last week. After the final immunization, the female mice were mated with male mice for two weeks. Offspring were obtained approximately 3 weeks after mating. All animal experiments were approved by the Institutional Animal Ethics Committee of the Wakayama Medical University (Project Number: 237 and 429).

### Division of offspring

In order to evaluate the importance of feeding status, we further divided offspring into 4 groups as follows. In Group A, offspring were delivered form PspA-immunized mother and breast-fed by their PspA-immunized mother. In Group B, offspring were delivered from sham-immunized mother and breast-fed by PspA-immunized mother. In Group C, offspring were delivered from PspA-immunized mother and breast-fed by sham-immunized mother. In Group D, offspring were delivered from sham-immunized mother and breast-fed by sham-immunized mother.

### Enzyme linked immunosorbent assay (ELISA)

Sera and milk were collected from mother mice at birth of their pups (day0), and on days 7, 14 days after their birth. Sera were also collected from offspring at birth (day 0), 7, 14 days after birth. Anti-PspA specific antibodies in milk and sera were evaluated by the solid ELISA. Briefly, 96 well microplates (MaxiSorp, Nunc, Roskilde, Denmark) were coated with 50 µl of rPspA (2 µg/ml) in phosphate buffered saline (PBS) overnight at 4°C. After washing three times with PBS containing 0.05% Tween 20 (PBS-T), the wells were blocked for 1 h with casein buffer (0.2% casein, 0.05% Tween 20 in PBS) at room temperature. Then, 50 µl of samples diluted with casein buffer were incubated at 4°C overnight. To determined PspA specific antibody isotypes, after washing with PBS-T, the plate was incubated with 50 µl of 1/3000 biotinylated antibody to mouse IgG, IgA or IgM (Southern Biotechnology Associates, Birmingham, AL, USA) diluted in casein buffer for 2 h at room temperature, respectively. Then, after washing with PBS-T, the plate was incubated with 1/4000 alkaline phosphatase conjugated streptavidin (Southern Biotechnology Associates) for 2 h at room temperature. Color was developed with p–nitrophenyl phosphate (PNPP) (Sigma Chemical Co., St. Louis, MO, USA) and the optical density of each well was measured by a spectrophotometer at 405 nm. The subclass of anti-PspA specific IgG antibody was also determined using 1/2000 biotinylated antibodies to mouse IgG1, IgG2a, IgG2b or IgG3 (Southern Biotechnology Associates).

### Challenge with pneumococci in nasal carriage, pneumonia, and bacteremia models

For nasal carriage, 7-day-old offspring were given the bacteria in a 5-µl volume of sterile Ringer's solution in a single nostril without anesthesia. Two days after inoculation, offspring were euthanized by CO_2_ inhalation and the nasal cavity of each offspring was washed by flushing 100 µl of Ringer's solution into the trachea and out through the nostrils. Next, the nasal tissue including nasal conchae, olfactory epithelium, and sinus mucosa was excised and were homogenized individually in 1 ml Ringer's solution as described previously [79].

For lung infection, the bacteria were given in a 10-µl volume of sterile Ringer's solution in a single nostril to 7-day-old offspring anesthetized with diethylether (Wako Chemical Co., Japan) to facilitate aspiration. After 3 days, offspring were sacrificed and the lungs were removed. The lobes of the lungs were placed into 1 ml of Ringer's solution in a stomacher bag and homogenized. Blood from the euthanized mice was also plated to determine numbers of CFU/ml. All specimens were serially diluted and plated on blood agar plates, blood agar plates supplemented with 4 µg/ml gentamicin, and blood agar plates supplemented with 4 µg/ml gentamicin and 5 µg/ml optochin. The viable pneumococcal counts were determined after overnight incubation.

For systemic fatal infection, 10-day-old offspring were given the bacteria in 0.1 ml sterile Ringer's solution intraperitoneally with anesthesia. The offspring were observed for 5 days to determine survival or the day of death.

### Statistics

The levels of PspA specific antibody in each group were compared by ANOVA test with Dunn's multiple comparison test. The IgG1/IgG2a ratio was compared by Kruskal-Wallis test with Dunn's multiple comparison test. The carriage density of challenged offspring in each group was expressed as log_10_ CFUs and compared by Kruskal-Wallis test with Dunn's multiple comparison test. Survival of challenged offspring in each group was assessed by Kaplan-Meier test with Log-rank test. Statistical values were calculated with Prism 4 (GraphPad Software, La Jolla, CA, USA). For all comparison, *p*<0.05 was considered to represent a significant difference.
